# Recommendations for performing measurements of apparent equilibrium constants of enzyme-catalyzed reactions and for reporting the results of these measurements

**DOI:** 10.3762/bjoc.19.26

**Published:** 2023-03-15

**Authors:** Robert N Goldberg, Robert T Giessmann, Peter J Halling, Carsten Kettner, Hans V Westerhoff

**Affiliations:** 1 Biosystems and Biomaterials Division, National Institute of Standards and Technology, Gaithersburg, Maryland, 20899, USA; 2 Institute for Globally Distributed Open Research and Education (IGDORE), Berlin, Germany; 3 University of Strathclyde, Glasgow G1 1XL, Scotland, UK; 4 Beilstein-Institut, Trakehner Straße 7–9, 60487 Frankfurt am Main, Germany; 5 Swammerdam Institute for Life Sciences, Faculty of Science, University of Amsterdam, 1098 XH Amsterdam, The Netherlands; 6 Molecular Cell Biology, Faculty of Science, Free University Amsterdam, The Netherlands; 7 School of Biological Sciences, Faculty of Biology, Medicine, and Health, University of Manchester, Manchester, UK

**Keywords:** enzyme-catalyzed reactions, equilibrium constant, standards, thermodynamics

## Abstract

The measurement of values of apparent equilibrium constants *K*′ for enzyme-catalyzed reactions involve a substantial number of critical details, neglect of which could lead to systematic errors. Here, interferences, impurities in the substances used, and failure to achieve equilibrium are matters of substantial consequence. Careful reporting of results is of great importance if the results are to have archival value. Thus, attention must be paid to the identification of the substances, specification of the reaction(s), the conditions of reaction, the definition of the equilibrium constant(s) and standard states, the use of standard nomenclature, symbols, and units, and uncertainties. This document contains a general discussion of various aspects of these equilibrium measurements as well as STRENDA (**St**andards for **R**eporting **En**zymology **Da**ta) recommendations regarding the measurements and the reporting of results.

## Perspective

### 1. Introduction and motivation for recommendations

The aim of the STRENDA (**St**andards for **R**eporting **En**zymology **Da**ta) Commission [1] is to establish standards for reporting the results of measurements related to enzymology with the aim to improve the quality and the findability, accessibility, interoperability, and reliability (FAIR Data Principles) [2] of data published in the scientific literature. Equilibrium constants *K* and apparent equilibrium constants *K*′ comprise an important set of physiochemical property data and are essential for the calculation of the direction and extent of reaction and the concentrations of species in complex reaction mixtures at equilibrium. Thus, values of *K* and *K*′ are used to determine the practicality of using a reaction to manufacture a substance and for process optimization in bioengineering applications. These values can also be used in the analysis of the kinetics of enzyme-catalyzed reactions and to gain insight into the operation and modeling of metabolic pathways [3] and genome wide networks [4], particularly if one has limited in vivo measurements of the substances in the pathway [5–6]. However, we have observed that a fair number of investigators overlooked important aspects of their measurements and/or failed to report valuable information [7]. Not only can these apparently minor deficiencies lead to accumulating errors in the analysis of metabolic networks, they also lead to what has been called the irreproducibility crisis [8]. Indeed, the performance and reporting of equilibrium measurements involves a number of subtle but important matters some of which can be easily missed, particularly by investigators new to this measurement. Thus, the STRENDA working group saw the need for a careful discussion on the design and execution of equilibrium measurements on enzyme-catalyzed reactions with emphasis on avoiding possible systematic errors and having a thorough reporting of results.

This document is an addendum to the STRENDA Guidelines [9] which must be kept brief and thus benefit from supplementary information. These recommendations incorporate the recommendations made in 2011 by the International Union of Biochemistry and Molecular Biology (IUBMB) [10]. The principal author of these IUBMB recommendations was the late Robert A. Alberty, who was also instrumental in formulating the Legendre transform thermodynamic formalism, thereby legitimizing the use of the apparent equilibrium constant *K*′ and the standard transformed molar Gibbs energy of reaction (Δ_r_*G*'°) for biochemical reactions [11]. STRENDA does not aim at policing authors who report data on enzyme kinetic or on equilibrium measurements. The aim is to aid these researchers by providing conceptual and practical guidance, comprehensiveness, and completeness of reporting of results and to aid readers to corroborate, analyze, and use the results for future applications and investigations.

### 2. Thermodynamic background

Firstly, one must recognize and make clear the difference between equilibrium constants *K* and apparent equilibrium constants *K*′ as well as between “chemical” and ”biochemical” reactions. Both chemical and biochemical reactions are carried out at fixed temperature *T* and pressure *p*. A chemical reaction involves species (often ionic) in specific protonation states and keeps track of the number of hydrogen ions produced (or consumed) in the reaction. Thus, if protons are produced or absorbed, the pH will change. A chemical reaction equation must balance all elements including hydrogen, magnesium, and calcium as well as electric charge and has an equilibrium constant denoted by *K*. An (overall) biochemical reaction involves sums of species that differ only in protonation and, if present, Mg^2+^ and Ca^2+^ binding states. It has an equilibrium constant *K’*, which is referred to as an “apparent equilibrium constant” [10]. A biochemical reaction should be carried out at essentially a constant pH and ionic strength, and, if Mg^2+^ or Ca^2+^ are involved in the reaction, at constant pMg and pCa. Consequently, the apparent equilibrium constant *K’* is defined in terms of total (sum) concentrations and it depends on pH, pCa, pMg, and ionic strength *I*, whereas the equilibrium constant *K* defined in terms of activities does not. The single biochemical reaction equation should not show individual charged and bound species, i.e., only sums of species for each biochemical reactant should be shown in the reaction equation. Atoms of C, N, O, P, S, and other atoms (but not H, Mg, or Ca) are included in the conservation matrix for a biochemical reaction equation and these atoms are conserved. Thus, since the pH is constrained by the use of a buffer, hydrogen atoms are not included in the conservation matrix [10]. Similarly, as pMg or pCa are constrained (i.e., constant pMg or pCa), they, too, are not in the conservation matrix. Consequently, chemical and biochemical reactions have distinctly different physical and chemical bases. Therefore, they must not be confused, intermingled, or combined under any circumstances. And, one must be able to distinguish between these two types of reactions on sight. Corresponding to a single biochemical reaction, there could be a large number of chemical reaction equations, one for each combination of the chemical species involved in the system of reactions and in any possible association state with H^+^, Mg^2+^ and Ca^2+^.

An example of the distinction between a biochemical reaction and the many chemical reactions that accompany it is the hydrolysis reaction of ATP (adenosine 5’-triphosphate) to {ADP (adenosine 5’-diphosphate) + phosphate}:


[1]

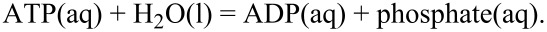



The apparent reaction quotient for this reaction is


[2]

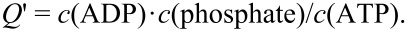



And the apparent equilibrium constant is


[3]

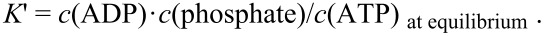



Here, (aq) denotes that the reaction is taking place in aqueous media and *c* denotes the respective concentrations (units of mol∙dm^−3^) of total amounts of ATP, ADP, and phosphate. Note that *Q*′ and *K*′ have the same form but they differ *critically* in that a measured value of *K*′ refers to Equation 1 having been established to be at equilibrium while a measured value of *Q*′ has not (yet) been established to be at equilibrium. Thus, at equilibrium, the measured value of *Q*′ is equal to *K*′. An important point made in these recommendations (see section 3.9) is that one must establish that the reaction under investigation has, in fact, reached equilibrium. The total ATP concentration is the sum of the concentrations of the individual ionic species formed by the protonation or metal ion binding reactions of the ATP species


[4]

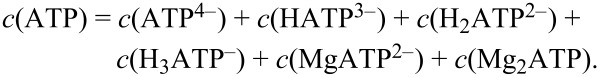



Additional metal ion complexes (e.g., involving Ca^2+^, K^+^, and Na^+^) can also be included in Equation 4. Similar expressions can be written for total ADP and total phosphate. The word “total” is sometimes used to specify total concentrations and, while useful, it seems cumbersome and unnecessary if the context is clear. A substantial number of chemical reference reactions can be selected for the biochemical Equation 1 – a few examples are


[5]

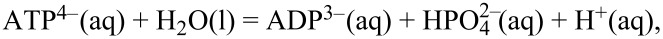




[6]

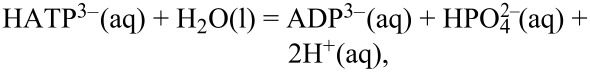




[7]

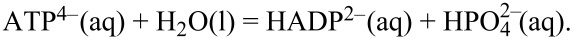



Note that different numbers of protons are shown on the right-hand side of Equations 5, 6, and 7. Thus, the change in binding Δ_r_*N*(H^+^) = 1 for Equation 5, but Δ_r_*N*(H^+^) = 2 and 0 for Equation 6 and Equation 7, respectively. The overall biochemical Equation 1 is, in fact, an ensemble of many individual chemical reactions. And, the value of Δ_r_*N*(H^+^) for the (overall) biochemical reaction can be calculated by using appropriate weighting factors for the values of Δ_r_*N*(H^+^) for each chemical reaction in this ensemble of chemical reactions. Thus, in general, Δ_r_*N*(H^+^) does not have an integral value. Corresponding to each of these chemical reactions, there is an equilibrium constant *K*. Thus, for Equation 5


[8]

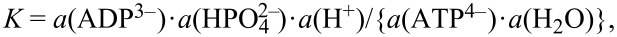



where *a* is the activity of the specified species. Note that the determination of *K*′ relies on measured total concentrations and should be reported without any corrections for activity coefficients. Thermodynamic methods [12–13] can be used to calculate values of *K*′ if one knows the values of *K* for a chemical reference reaction and for the protonation and metal ion binding reactions of the ATP, ADP, and phosphate species. These calculations require activity coefficients (see section 3.6) which account for both long-range electrostatic interactions and the interactions of the ions with each other and with the solvent. The inverse calculation to obtain *K* from *K*′ can also be performed by using regression. The scientific principles behind these calculations and transformed thermodynamic quantities are discussed in reference [11].

Also, it is important to recognize that the Gibbs energy *G* and the transformed Gibbs energy *G*′, corresponding to chemical and biochemical reactions, respectively, are state functions. Therefore, values of equilibrium constants and apparent equilibrium constants, respectively, are both a property of the reaction itself and are independent of the catalyst used. Indeed, there are some enzyme-catalyzed reactions that will proceed without an enzyme, e.g., carbonic anhydrase or at an elevated temperature. At equilibrium, the molar Gibbs energy of a chemical reaction Δ_r_*G* equals zero. Consequently, the standard molar Gibbs energy change Δ_r_*G*° = −*RT*log_e_*K*, where *R* is the gas constant and *K* is the equilibrium constant written in terms of activities *a* of chemical species. An activity equals the concentration (i.e., molality *m*, concentration *c*, or mole fraction *x*) multiplied by an activity coefficient *γ*, which is a function of the ionic strength and temperature. The transformed molar Gibbs energy of a biochemical reaction Δ_r_*G*′ also equals 0 at equilibrium and the standard transformed molar Gibbs energy change Δ_r_*G*′° = −*RT*log_e_*K*′, where *K*′ is written in terms of the ratio of the concentrations of the reactants at equilibrium, where each of the latter is the sum of a number of species differing in proton, Mg, and Ca binding. Note that no adjustment for activity coefficients is made to *K*′ and its value of *K*′ pertains solely to what has been measured. Whenever, the chemical species involved in a reaction bind to H^+^, Mg^2+^ or Ca^2+^, the two types of equilibrium constants, *K* and *K*′, are distinctly different physical quantities and, in general, have different values.

### 3. Recommendations

#### 3.1. Identification of substances and sources of materials

The identity of the principal substances used in the investigation should be stated unequivocally. This can be accomplished by use of standard (e.g., IUPAC) and commonly accepted names, one or more identifiers (Reaxys, PubChem CID, ChEBI ID, CAS, or InChI), and, *most importantly*, by presenting the structures of the reactants and products in a form that shows the positions of all of the atoms in a substance. This could be a picture, but would preferably be an MDL Molfile [14] that includes accurate, 3-D positions of all atoms in the molecule. The last method is, by far, the most definitive method and avoids causing the reader to consult the literature to obtain the structure(s) of the substances used as well as avoid possible confusion regarding substance identification. A combination of the aforementioned methods is recommended. If substances have chirality, attention to which chiral forms are present is also required. The enzyme(s) used in a study should be clearly identified, e.g., by giving the UniProtKB [15] and/or Protein Data Bank [16] identifier(s) and origin (e.g., species, tissue). If the enzyme has not been registered, one should provide as much information as possible, i.e., the source and the amino acid sequence. Reporting an Enzyme Commission number [17] is also helpful. If a recombinantly expressed enzyme is used, the intended amino acid sequence of the enzyme should be reported. Many biochemical substances exist as a multiplicity of species in aqueous solution (e.g., ATP is a mixture of ATP^4−^, HATP^3−^, H_2_ATP^2−^, MgATP^2−^, etc.). In addition to the total concentration of ATP in all of its bound or liganded forms, one should report the unambiguous identifier of the substance that was actually used in the study. The identifiers can be a CAS number, a PubChem CID, ChEBI ID, or any database entry (e.g., adenosine 5′-triphosphate, disodium salt hydrate, CAS number 34369-07-8) along with the concentrations at which it was used.

The sources of all substances used, their batch numbers, and estimated purities, as well as the methods of analysis used by the manufacturer and by the investigators should be reported. Also, since water is present as an impurity in most substances and since weighing is often used to determine the amounts of substances, the moisture content of the principal substances used in a study should be reported. If the moisture content is important and can vary, one should report the method used to control and measure the amount of moisture. Since the relative molecular masses *M*_r_ are used to convert masses of substances to concentrations or molalities, the values of *M*_r_ should be reported for all of the substances used in the study. The reference(s) for the used values of *M*_r_ and the underlying relative atomic masses *A*_r_ should be reported.

#### 3.2. Description of equipment and procedures

It is essential that a complete description of the equipment and procedures be reported along with a complete reporting of the results of the measurements. While graphical presentation is useful to show trends, the presentation of numbers in tabular form is critical. It is helpful to the reader, and in compliance with the FAIR guiding principles for scientific data management and stewardship [2], if all quantities are defined in the data table itself and if this table is in a computer readable form, e.g., in a txt (comma or semi-colon-separated), PDF, or Excel format.

#### 3.3. Standard nomenclature, symbols, and units

Results for all property values should be reported by using internationally accepted names for quantities and symbols and SI units [10,18].

#### 3.4. Specification of the reaction

One must specify the reaction that has been studied, the stoichiometries, the units used for concentrations, and the solvent. The basis for the stoichiometry should be reported for a binding reaction. If substances such as CO_2_, N_2_, and NH_3_ are reactants, one must specify if these substances are in solution or in the gas phase and give the units used. All important aspects of the reactants, the reaction, and the catalyst should be included in the discussion, e.g., if reactants are bound to a membrane or surface or in equilibrium with a solid.

#### 3.5. Definition of equilibrium constants and specification of standard states

Attention to standard states must be paid when water is a reactant. One generally takes the activity (or concentration) of water to be unity [19] when calculating the equilibrium constant for a reaction that occurs when water is the solvent and is present in vast excess. However, biochemical reactions can also take place in non-aqueous solvents and with water as a reactant. In such cases, one must measure the concentration of water in the non-aqueous phase in order to be able to calculate rigorously a value for the equilibrium constant. And, per section 2, the equilibrium constant must always be clearly defined. Thus, for studies involving homogeneous non-aqueous media, the water concentration is best obtained by a direct measurement, e.g., a Karl Fischer titration [20]. When there are two or more phases present, one should specify the phase in which the water concentration has been measured and whether the water is assumed to have equilibrated across the phases. If the concentration of water in the non-aqueous phase cannot be measured, there is still a utility in having the concentrations and/or ratios of the other reactants.

When reporting the value of an equilibrium constant, particularly for an unsymmetrical reaction (i.e., a reaction where the number of molecules on the left-hand side of the reaction differ from the number of molecules on the right-hand side), one must specify the units of concentration used to calculate the equilibrium constant. It is recommended that the standard state [19] be based either on 1 mol∙kg^−1^ (molality) or 1 mol∙dm^−3^ (concentration). There must be no ambiguity in the direction of the reaction to which the value of *K* or *K*′ pertains. Thus, writing the reaction equation itself is essential. And one should give the equation that defines *K* or *K*′, with the equilibrium constant defined in the usual way as the arithmetic product of the concentrations of the substances/species on the right-hand side of the reaction equation (the products) to the respective power of their stoichiometric numbers divided by the arithmetic product of the concentrations of the substances/species on the left-hand side of the reaction equation (the reactants) to the respective powers of their stoichiometric numbers [18]. Since one can also use activities, molalities, and mole fractions to define the equilibrium constant [18], it is *critical* to specify exactly how the equilibrium constant is defined along with the standard state that is used. Since activities are often used for the equilibrium constant *K*, one must state the basis or source for the activity coefficients that have been used. Note that *K*′ is defined only in terms of concentrations, molalities, or mole fractions, not activities [11].

#### 3.6. Specification of constraints, conditions of reaction, pH, and activity coefficients

The measurement of *K*′ is generally done by measuring concentrations of reactants and products and then calculating the apparent reaction quotient *Q*′. As stated earlier, *Q*′ is defined as a ratio of concentrations and is similar in form to *K*′. However, and most importantly, one cannot state that *K*′ is equal to *Q*′, until one has established that the reaction is at equilibrium. Ideally, one measures the concentrations of all reactants and products. However, this is not always practical and one often relies on the measurement of at least one reactant or product to obtain the extent of reaction *ξ*′ for the biochemical reaction.

The use of a buffer for H^+^ introduces a constraint in that the activity of H^+^(aq) is held constant in the reacting system. Similarly, it is possible to introduce constraints on other reactants such as Mg^2+^ or Ca^2+^ by use of a suitable buffer (e.g., EDTA) for either of these ions. Also, if one keeps the concentration of one of the reactants in a very substantial excess above the concentrations of the other reactants, the concentration of that reactant is effectively a constraint on the reaction. Then, at equilibrium, the concentration of that reactant is known from its initial concentration with, perhaps, a small correction. All constraints on the reacting system and all auxiliary data used should be reported. The pH that should be reported and used in all calculations is the pH at equilibrium.

Both concentration *c* (units of mol·dm^−3^ or amount of a substance in 1 dm^3^ = 1 L of solution) and molality *m* (units of mol∙kg^−1^ or amount of a substance in 1 kg of solvent) are widely used. Molality has the advantage that its value does not change with temperature and it is easily calculated from laboratory determinations of mass. Thus, if one uses molality based values of *K* when calculating the value of a standard molar enthalpy of reaction Δ_r_*H*° from values of *K* as a function of temperature *T*, one avoids the need for a correction due to the temperature dependence of the density of the solvent. Concentration expressed as mol∙dm^−3^ has important advantages when dealing with transport properties (e.g., diffusion coefficient and transport number). In this document the word “concentration” is used with the understanding that “molality” or mole fraction could also have been used.

The reaction equation and the values of the experimental parameters under which the reaction has occurred must be reported. Accordingly, one must measure and report the temperature *T*, the pressure *p*, the pH, the concentrations of all of the substances in solution, and any other quantities that are relevant to an unambiguous statement of what was done in the measurement. Clearly, periodic calibration of all equipment is essential. It is advised to use standard buffers to calibrate the pH meter that is being used in a study. The standard buffer(s) used should have p*K* values near the measured pH value(s). As stated above, in many investigations the measured change in concentration of a single reactant or product is used to obtain the extent of reaction *ξ*′. The concentrations of the other substances are then calculated by using their initial concentrations and *ξ*′. While it is desirable to measure the concentrations of as many of the reactants as possible, this is not always practical. Thus, it is important to check for side reactions. The pMg and the ionic strength are presently not measured directly but should be calculated if at all possible [13]. To do this, one must know the relevant proton and metal-ion binding constants along with the concentrations of all of the substances in the solution. This calculation also requires either a knowledge of or estimated values of the activity coefficients *γ*_i_ of the species involved in the reactions occurring in the solution. The *γ*_i_ values are also needed to calculate activities *a*_i_ from measured or calculated concentrations. This accounts for the effects of the ionic strength on the equilibrium constants in the reacting system. It is recommended that the experimental quantities, i.e., *T*, *p*, pH, and the concentrations of all of the substances in solution be reported. One should also calculate pMg and *I* and report these values. A more extensive equilibrium modeling calculation [11–12,21] can be performed that will lead to values of the equilibrium constant *K* and the standard molar enthalpy of reaction Δ_r_*H*° for a chemical reference reaction that involves specific ionic species and that is one of the chemical reactions that comprise the ensemble of reactions that make up the (overall) biochemical reaction. In terms of experimental design and to reduce the complexity of the calculations, it is recommended that reaction conditions are robust with respect to changes in pH, pMg, pCa, and *I* due to reaction progress. This can be accomplished by use of sufficiently strong buffers for H^+^, Mg^2+^, and Ca^2+^ and by keeping *I* essentially constant.

As mentioned above, values of activity coefficients *γ* are needed to calculate values of *K* and Δ_r_*H*° for a chemical reference reaction from measured values of *K*′. In most cases, these values of *γ* are not available from measurements. Thus, Alberty et al. [10] used an extended Debye–Hückel equation


[9]

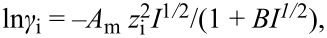



where *γ*_i_ is the activity coefficient of species i, *A_m_* is the Debye–Hückel constant (see [22] for values of Debye–Hückel constants from 0 °C to 150 °C), *z*_i_ is the charge number of ion i, and *B* is a constant which is often referred to as the “ion-size” parameter. Alberty et al. [10] used the value *B* = 1.6 kg^1/2^∙mol^−1/2^ based on the values of this parameter obtained in fitting data on a series of electrolytes of charge type 1-1, 1-2, and 2-1 [23]. The extended Debye–Hückel theory may not be accurate for *I >* 0.1 mol∙dm^−3^, which is well below the physiological ionic strength of 0.25 mol∙dm^−3^ where *K*’ is frequently measured. However, the afore mentioned procedure for estimating values of activity coefficients offers a standard way of dealing with the ionic strength and the option of back calculation if better approaches become available. It is desirable to have values of *K*′ at various ionic strengths which would allow for extrapolation to *I* = 0.

#### 3.7. Specification of chemical reference reaction, near physiological conditions, and thermodynamic calculations

The choice of the chemical reference reaction out of the set of chemical reactions connected by H^+^, Mg^2+^ or Ca^2+^ association or dissociation reactions, is arbitrary. If possible, it is recommended that this calculation of *K* and Δ_r_*H*° be performed from *K*′ and Δ_r_*H*′°, respectively, even if the focus lies on the apparent equilibrium constant *K*′. If this calculation is done, the method of calculation should be described and any auxiliary data or assumptions used in these calculations should be reported.

The following have been widely used as near physiological conditions: *T* = 310.15 K, pH = 7.0, pMg = 3.0, and *I* = 0.25 mol∙dm^−3^ [10]. However, since there is no unique set of physiological conditions, it may be necessary to choose conditions other than the aforementioned ones. If possible, the measurement of a value of *K*′ under the above “standard” conditions, will always be an asset. And, having values of *K*′ under a variety of conditions is also useful. In particular, a variation of *T* and pH can significantly increase the applicability of the results to other relevant physiological conditions. A recommended standard medium is given by van Eunen et al. [24]. There is substantial scientific value gained from having a reliable measurement of *K*′ for a biochemical reaction under a specific set of conditions. The value of such an investigation is enhanced by having additional measurements of *K*′ at various values of *T,* pH, pMg, and *I*. If *K*′ is known at one temperature, Δ_r_*G*′° can be calculated at that temperature. But, if *K*′ is measured at different temperatures one can also calculate the standard transformed molar enthalpy of reaction Δ_r_*H*′° and then the standard transformed molar entropy of reaction Δ_r_*S*′°. Using Δ_r_*H*′° one may then calculate Δ_r_*G*′° and *K*′ at different temperatures. Additionally, a measurement of the calorimetrically determined molar enthalpy of reaction Δ_r_*H*(cal) can be used to calculate [10] the standard molar enthalpy of reaction Δ_r_*H*° for a selected chemical reference reaction and also the standard transformed molar enthalpy of reaction Δ_r_*H*′° for the overall biochemical reaction. Measurement of values of both Δ_r_*H*(cal) and of *K*′ at different temperatures allows for a check on the accuracy of the results obtained from both calorimetric and equilibrium measurements. Note, however, that Δ_r_*H*′° and Δ_r_*H*(cal) differ by the product of the enthalpy of protonation of the buffer Δ_r_*H*°(buffer) and the change in binding of H^+^(aq) in the biochemical reaction Δ_r_*N*(H^+^), i.e., Δ_r_*H*(cal) = Δ_r_*H*′° + Δ_r_*N*(H^+^)∙Δ_r_*H*°(buffer) [10]. Analogous equations pertain should a Ca^2+^ or Mg^2+^ buffer be used.

#### 3.8. Interferences

Appropriate control experiments should be performed to make certain that the enzyme or, if it is in a suspension or solution, that an impurity in the enzyme suspension or solution does not interfere with the measurement. Thus, when a spectroscopic method is used for an equilibrium measurement (e.g., in an NAD/NADH coupled reaction), it has been customary to perform a control experiment in which the enzyme is added to the buffer to determine if there is a change in absorption. Clearly, if a change is observed, a correction must be applied. Note that if the enzyme is removed prior to the measurement of concentrations (e.g., filtration or use of a guard column in an HPLC), the enzyme itself should cause no interference with the measurement. Since interferences may also be caused by light scattering if the measurement is spectroscopic, centrifugation prior to measurement may be needed. However, it is good practice in any measurement to perform a control experiment to make certain that there is no interference from the enzyme or from the solution in which the enzyme may be situated.

#### 3.9. Definition and establishment of chemical equilibrium

Loss of enzyme activity due to product inhibition and thermal instability can lead to a failure to achieve equilibrium and consequently large systematic errors in the measured values of equilibrium constants (both *K* and *K*′) for enzyme-catalyzed reactions. This matter requires a careful consideration in regards to the definition of chemical equilibrium, the approaches to equilibrium, and the various methods used to measure *K* and *K*′. Firstly, we shall assume that a state of equilibrium exists when the forward and reverse reactions proceed at the same rate. This occurs at a microscopic level and while the macroscopic concentrations of the reactants do not change at equilibrium, the system is not static on a microscopic level. Thus, a double arrow (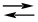) is sometimes used to denote a state of equilibrium for a reaction. And, the necessary macroscopic criteria for a demonstration that equilibrium has been achieved are that (1) the value of the reaction quotient *Q* does not change with time and (2) motion towards that value of *Q* has been demonstrated by a change in the value of *Q* from opposite directions of the reaction. This constitutes the operational definition of chemical equilibrium that is used in our discussion of the various methods used to measure equilibrium constants of enzyme-catalyzed reactions. Note that the aforementioned criteria cannot be met if the enzyme is not active. Also, the presence of side reactions in the reaction mixture is not a problem as long as the reaction of interest is at equilibrium. In such cases, the substances in the side reaction must not interfere with the measurement and one must properly account for any changes in concentrations of the substances in the reaction of interest due to the side reaction(s). In our discussion of the various methods used to measure equilibrium constants, we will use only the symbols *K* and *Q* with the understanding that the discussion also refers to *K*′ and *Q*′, respectively*.*

A measurement that relies solely on the reaction quotient obtained from only one direction of reaction and a statement that the value of *Q* is not changing with time is insufficient evidence to establish that equilibrium has been achieved. The three rigorous methods that one can use for equilibrium measurements on enzyme-catalyzed reactions are now described.

Method 1: As a function of time, measure the approach to equilibrium from opposite directions of reaction, i.e., measure *Q*_F_ (forward direction) and *Q*_R_ (reverse direction). If the enzyme loses activity before the reaction reaches equilibrium, the result will be *Q*_F_ ≠ *Q*_R_. If the enzyme remains active, *Q*_F_ will be equal to *Q*_R_ within the experimental errors and *K* will be equal to the average of *Q*_F_ and *Q*_R_. The presence of side reactions and additional equilibria in solution will not affect the result of this type of measurement, unless the substances in the side reaction(s) interfere with the measurement of the concentrations of the substances in the reaction of interest or invalidate any assumptions regarding mass balance. Thus, the use of this method provides clear evidence that equilibrium has been achieved. However, unless the method used is convenient to perform (e.g., continuous spectroscopic monitoring), it requires a substantial amount of additional effort by the investigator. We note that it is not necessary to know the mechanism or the kinetic constants or the rate of approach to equilibrium to obtain an accurate value of *K*′. Nevertheless, the half-time to reach equilibrium can be estimated by assuming Michaelis–Menten kinetics. Thus, when the substrate and product concentrations are below their respective Michaelis constants (*K*_M_ and *K*_P_, respectively), one finds that


[10]

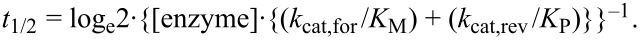



Here, [enzyme] is the concentration of the enzyme and *k*_cat,for_ and *k*_cat,rev_ are the catalytic rate constants for the forward and reverse reactions, respectively. The use of Equation 10 requires a knowledge of the Michaelis constants *K*_M_ and *K*_P_ (also called the product inhibition constant), *k*_cat,for, _*k*_cat,rev,_ and [enzyme]. Since this information is often not available, investigators have generally set the amounts of enzyme and substrates to use in their equilibrium measurements empirically (see section 3.13). However, Equation 10 can be used to help estimate the amount of enzyme needed to significantly increase (e.g., double) the rate of approach to equilibrium. Doing this is a good test to see if the measured value of *K*′ is independent of the amount of enzyme.

Method 2: Approach the position of equilibrium from two different directions, i.e., add enzyme to two solutions at the start of the experiment. One of these solutions contains only the reactants on the left side of the reaction equation and the second solution contains only the reactants on the right side of the reaction equation. After waiting a suitable period of time, measure the reaction quotient *Q*_F_ from the forward and *Q*_R_ from the reverse directions of reaction. Measurements that demonstrate the approach to equilibrium are not performed. This is the most commonly used method for the measurement of *K*. But let us examine this method in detail and in regards to the matters of the possible loss of enzyme activity and the possibility of side reactions.

**Case I. The enzyme remains active.**

A. There are no side reactions. As above, if the enzyme loses activity before the reaction reaches equilibrium, *Q*_F_ ≠ *Q*_R_. If the enzyme remains active, *Q*_F_ will be equal to *Q*_R_ within the measurement uncertainties and *K* will be equal to <*Q*>, where the <> indicate the average of the values of *Q*_F_ and *Q*_R_^.^

B. There are side reactions occurring. If the rate of the side reaction(s) is faster or slower than the rate of the reaction of interest, the result that *Q*_F_ = *Q*_R_ is, in most cases, sufficient to state that *K* = <*Q*>.

However, caution is advised in some cases such as the hexokinase catalyzed reaction


[11]

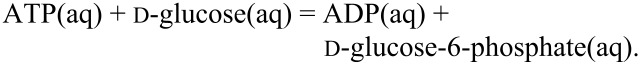



If, when measuring values of the apparent equilibrium constant for this reaction, there is also some glucose 6-phosphatase present, the following reaction will also occur


[12]

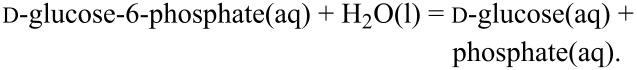



Thus, the production of ᴅ-glucose(aq) and the loss of ᴅ-glucose-6-phosphate(aq) via Equation 12 will affect the measured values of *Q*′ for Equation 11. These measured values of *Q*′ may be time dependent. Thus, the fact that *Q*′_F_ = *Q*′_R_ may be a coincidence and is not a complete demonstration that the measured <*Q*′*>* is equal to *K*′. In such cases, it is important to ascertain if there are any side reactions present that could cause this possible systematic error. In the specific example given, elimination of the glucose 6-phosphatase activity would be sufficient to avoid this error. If this is not possible, the use of method 1 where one measures the approach to equilibrium from opposite directions of reaction could be advantageous. Specifically, one can plot the values of *Q*′ for both Equation 11 and Equation 12 as a function of time and observe the approach to equilibrium. And, if *Q*_F_ = *Q*_R_ for the two respective reactions, both reactions are at equilibrium. Thus, all measured concentrations refer to equilibrium concentrations and the calculations of values of *K*′ for both Equation 11 and Equation 12 are rigorous. In the case that Equation 12 is not at equilibrium, one has still demonstrated that Equation 11 is at equilibrium. Clearly, in this particular use of method 1, one must have independent measurements of all concentrations and not rely on mass balance to obtain the concentrations. In any case, it is always useful to check for the presence of side reactions.

**Case II. The enzyme is initially active but loses all activity after some time.** In many cases, equilibrium will not be reached and the result of the measurement will be that *Q*_F_ ≠ *Q*_R_. However, there is a possibility that the reaction reached equilibrium but that a side reaction has caused a change in the concentration of one of the reactants. And, if the change in concentration of that reactant is the same for both the F and R solutions, a systematic error would be made by taking *K* = <*Q*>. However, considering the very substantial range of values the true value of *K* could lie in and the improbability that the change in concentration due to a side reaction from both the F and R reaction mixtures is the same, it is unlikely that *Q*_F_ = *Q*_R_ should the aforementioned conditions be present. Nevertheless, a check should be made by adding additional *active* enzyme to either the forward or reverse reaction mixture (but not to both reaction mixtures) and, after a reasonable period of time, measuring the value of *Q* for that reaction mixture. If the value of *Q* has not changed, one has good evidence that equilibrium has been achieved. Note that while one might be tempted to also add additional substrate to one of the aforementioned reaction mixtures, doing this is not necessary as small fluctuations in the concentrations of reactants will occur as long as the enzyme is still active. The absence of side reactions would eliminate the aforementioned possible systematic error. Thus, a check on mass balance on side reaction products is recommended. If possible, it is best to measure the concentrations of all reactants. The use of modern mass spectrometry should make this possible for many reactions.

Method 3: Prepare several synthetic reaction mixtures having values of *Q*(initial) near the presumed value of *K* and then add the enzyme to each reaction mixture. After waiting a suitable and typically a relatively short period of time (maybe an hour or two), one then measures the change in the concentration of one or more of the reactants. By using mass balance, one can calculate *Q*(final). Then, for each reaction mixture, calculate Δ*Q* = *Q*(final) − *Q*(initial). The synthetic reaction mixture for which Δ*Q* = 0, contains the solution for which *K = Q*. While it is unlikely, that one of the initially prepared reaction mixtures meets this condition, one can plot Δ*Q* as a function of *Q*(initial) for the several synthetic reaction mixtures that were prepared and construct a curve that fits the data points on the plot. This curve must cross the abscissa in order to have a rigorous demonstration that equilibrium has been reached. And, if this is the case, the value of *Q* on the abscissa which corresponds to Δ*Q* = 0, yields the value of *K*. If any of the reactants are labile, this elegant method has the noteworthy advantage that equilibrium can be achieved in less time than approaching the position of equilibrium from the extreme forward and reverse directions. Also, it can be used with any type of measurement method and, in fact, calorimetry was used for this purpose in one early study [25]. A disadvantage is that its use requires some educated guessing as to the approximate value of the equilibrium constant. Nevertheless, this is a very powerful method for the measurement of equilibrium constants. It appears to have been used in only a few studies [25–28].

Note that the above discussion contains several subtle matters that can be missed by investigators. The bottom line is that the demonstration of equilibrium requires care and that systematic errors can be caused by failure to reach equilibrium, by side reactions, and by interferences. Also, all of the procedures for demonstrating that equilibrium has been achieved require the availability of the necessary substances so that equilibrium can be approached from opposite directions of reaction. If the necessary substances are not readily available, it is necessary to prepare them in order to perform a rigorous measurement of the equilibrium constant. Labile or difficult to obtain substances may be prepared in situ [29].

For reactions that proceed without the need of a catalyst, one can still raise the question of whether or not a given reaction has reached equilibrium. In such a case, the above discussion holds except that, in all of the methods discussed above, there is no need to add a catalyst. In particular, for the case where one has approached equilibrium from both directions of the reaction, one does not need to perform the control experiment that involves the addition of the catalyst (enzyme) after the reaction has presumably reached equilibrium. NMR and radioactive exchange methods can be used to ascertain that, in fact, the reaction of interest has occurred at a microscopic level and from both directions of reaction. Binding, ionization, and reactions that involve the formation of complexes often proceed rapidly. In such cases, the assumption is generally made that equilibrium has been achieved. In any case, any assumption regarding the attainment of equilibrium should be made explicit when reporting results.

#### 3.10. Calorimetric measurements

If calorimetric measurements are performed, it is important to establish the accuracy of the calorimeter used [30] and to measure the extent of reaction. If there are any side reactions, one must apply a correction to the measured enthalpy change Δ*H* for the Δ*H* values due to these side reactions. Necessary control experiments and corrections are the measurement of (1) Δ*H* for the mixing of the buffer solution that contains the enzyme with the buffer used and (2) Δ*H* for the mixing of the solution that contains the substrates in buffer with the buffer itself. Note that the calorimetrically determined molar enthalpy of reaction Δ_r_*H*(cal) requires a correction for the molar enthalpy of protonation of the buffer present [10] if one wishes to calculate Δ_r_*H*°. To perform this correction, one needs a value for Δ_r_*N*(H^+^) and the standard molar enthalpy of protonation of the buffer [10]. A value for Δ_r_*N*(H^+^) can be obtained by using equilibrium modeling calculations. Alternatively, Δ_r_*N*(H^+^) can be measured by using a spectroscopically detectable pH indicator or a calibrated pH electrode. These latter measurements require substantial sensitivity, perhaps a reduced buffer concentration, and great care to obtain an accurate value of Δ_r_*N*(H^+^). The magnitude of this correction can be minimized by using a buffer that has a small absolute value for Δ_r_*H*°(buffer), e.g., a phosphate buffer. The aforementioned buffer protonation correction is not needed for standard molar Gibbs energy changes calculated from the equilibrium concentrations nor for standard molar enthalpy changes calculated from the temperature dependence of the equilibrium constant. Equilibrium modeling calculations can also be used to predict how *K*′ and Δ_r_*H*′° vary with *T*, pH, pMg, and *I* [21].

#### 3.11. Comparisons with values from the literature, network calculations, and the use of other methods

A standard part of reporting any measurement is to make a comparison of the value(s) obtained in the current study with previously reported values of the measured quantity in the scientific literature. Thus, comparisons with previously measured values of *K* or *K*′ of the same or highly similar reactions should be made and reported. In thermodynamics, the measured quantities are frequently related to the Gibbs energy *G*, the enthalpy *H*, and the entropy *S*. The aforementioned quantities are state functions and are independent of pathway. Therefore, in a fair number of cases, one can find thermochemical pathways that allow for an alternative way to calculate the measured property values of interest. These pathway calculations are also referred to as thermodynamic network or thermodynamic cycle calculations. It is recommended that the use of this approach be considered and, if the necessary data are present in the literature, that the thermodynamic pathway calculations be performed. Details regarding thermodynamic network calculations for biochemical substances are given in reference [31] together with references to publications that contain the results of these calculations and that also give the values of standard molar formation properties. Also, comparisons of the measured property value(s) can sometimes be made with property values for other substances and reactions that involve the same or a similar change in chemical bonding, e.g., by using group contribution methods [32–35]. And, one should not overlook the use of computational chemistry as a means to obtain the desired property values. Finally, there is a close relationship between equilibrium constants and certain enzyme kinetics parameters via the Haldane relationships. These relationships are discussed in references [10,36] and, in fact, values of *K*′ have been obtained for a fair number of enzyme-catalyzed reactions by measuring rates of reaction and obtaining the enzyme kinetic parameters [10].

#### 3.12. Uncertainties

A proper assessment of the uncertainty of a measured property value must consider both random and possible systematic errors. Guidance on this matter is given in the International Standards Organization’s “Evaluation of measurement data – Guide to the expression of uncertainty in measurement” [37]. In view of the increased attention to irreproducibility [8,38] in the sciences it is advisable to ascertain the reproducibility explicitly. This can be done by having the experiments repeated by different staff members and*, if possible*, by using different methods and in different laboratories (e.g., see reference [39]), and, in particular, by a careful consideration of possible systematic errors [8]. As mentioned above, a further check on the values of measured property values can sometimes be obtained by using a thermodynamic pathway calculation. An independent assessment of the published information, e.g., peer review, is, of course, necessary.

#### 3.13. Additional considerations: methods of analysis and the use of the enzyme

There are several practical matters that play into any experimental investigation. One is the choice of an analytical method. Enzymatic assays, spectroscopic methods, and HPLC have proven to be workhorses in much of the literature in this area. Spectroscopic absorbance has proven to be exceptionally useful for reactions involving nicotinamide adenine dinucleotide and nicotinamide adenine dinucleotide phosphate. Radioactivity and GC methods have been used occasionally. NMR and mass spectrometry can also be used in equilibrium investigations. In fact, essentially any analytical method might, in principle, be made to work. But, each analytical method has possible systematic errors associated with its use. For example, if a value of a molar decadic absorption coefficient (extinction coefficient) is used, one must either trust the value obtained from the literature or use a reliable method to measure its value, e.g., NMR [40–41]. If the enzyme used in a study binds to one or more substrates, one must determine whether this interferes with the measurement of the substrate concentrations. One can minimize this error by keeping the enzyme concentration less than one or two percent of the concentrations of the substrates. Also, by using a guard column or centrifugation, one can remove the enzyme prior to analysis and avoid this possible error.

The amount of enzyme to use in an investigation may be estimated by knowing both the activity (either on a massic or on a volumetric basis) of the enzyme and how the activity changes with reaction conditions such as pH, *T*, *I*, and pMg. Some purification of the enzyme may be required to avoid side reactions or interfering impurities. Knowledge of the stabilities of the enzyme and the substrates can also aid with the design of the measurement. This must often be obtained empirically. Varying the amount of enzyme is indeed a good test to see if the measured values of *K*′ are independent of the amount of enzyme.

In the case where a reaction proceeds overwhelmingly in a single direction, there will be only a very small amount of the reactant(s) on one side of the reaction equation remaining at equilibrium. Thus, an accurate measurement of the concentration of only one of these reactants (i.e., the one having a very low concentration) may be sufficient for the determination of an accurate value of *K*′ for the reaction of interest.

A difficulty can arise if a substrate is not readily available. This is a serious problem as the operational definition of chemical equilibrium (see section 3.9) *requires* that one must demonstrate the approach to equilibrium from different directions of reaction. Thus, as mentioned in section 3.9, some investigators have used very small quantities of a substrate that required substantial synthetic efforts [25] or have prepared the needed substrate in situ [27].

#### 3.14. Importance of careful reporting

IUPAC has published a “Guide to the Procedures for the Publication of Thermodynamic Data” [38] and CODATA has published a “Guide for the Presentation in the Primary Literature of Numerical Data Derived from the Experiments” [42]. Both of these Guides provide useful information on the reporting of physical property data. The importance of reporting essential information and results was emphasized in the 1972 IUPAC Recommendations [43]: “The highly interdependent nature of thermodynamic data imposes special obligations upon the author of papers reporting the results of thermodynamic investigations. She/he must give enough information about her/his experiment to allow readers to appraise the precision and accuracy of their results so that they may be properly consolidated within the existing body of data in the literature. Further, as accepted values of physical constants change or as new thermodynamic data for related systems become available, subsequent investigators often can recalculate results if it is clear that they are based on good experiments for which adequate and sufficiently detailed information is presented, however old they may be. For these reasons, an author's prime responsibility is to report his/her results in a form related as closely to experimentally observed quantities as is practical, with enough experimental details and auxiliary information to characterize the results adequately and to allow critical assessment of the accuracy claimed. For the convenience of the reader, the author may interpret and correlate the primary results as appropriate and present derived results in a form easy to utilize. However, such derived (or secondary) results never should be published at the cost of omitting the primary results on which they are based. Reference may be made to accessible earlier publications for some details.” Clearly, the earlier publications(s) should be checked to be certain that they lead to complete and explicit information. The general advice given in the 1972 IUPAC Recommendations have been systematized and detailed in terms of nine general principles in a more recent IUPAC publication [44]. Indeed, the analysis of laboratory data and the careful reporting of results often take an amount of time comparable to the time spent in performing the measurements. However, such studies where this has been done will have lasting archival value.

## Summary

This document contains STRENDA recommendations regarding the measurement and reporting of apparent equilibrium constants *K*′ for biochemical reactions. It is important to note that there are several subtleties associated with these measurements and that there are several details that could be missed even by experienced investigators. Thus. the STRENDA working group believes that these recommendations will serve as an aid to those performing equilibrium measurements. However, it is not our intention to make perfection the enemy of the good. Indeed, an investigator who has reported a new enzyme or a variant of one may simply wish to demonstrate the reversibility of the reaction and to report an approximate value of *K*′ together with the experimental conditions. A similar circumstance is a chemical engineer using an enzyme-catalyzed reaction for a manufacturing application and who also reports a value of *K*′. In such circumstances, only the relevant experimental quantities and conditions (i.e., the pH, *T*, and concentrations of the substances in solution at equilibrium) are important to report and a complete analysis of the thermodynamics in terms of all ionic species that may be involved in the reaction and the corresponding calculation of the equilibrium constant *K* for the chemical reference reaction would be above and beyond what is customarily done in such studies. Thus, reporting of the value of *K*’ defined in terms of total concentrations is sufficient. If the authors are interested in wider applications and thereby higher impact of their work, the next recommendation is that they also determine how *K*′ varies with *T*, pH, pMg, and *I*. Doing this would allow users to estimate the chemical equilibrium constant for the component chemical reactions and the *K*’ for experimental conditions that suits them better. Even more interested users may then use values of p*K* and Δ_r_*H*° to calculate values of *K*’s of the chemical reactions that constitute the overall biochemical reaction. In all cases, a demonstration that equilibrium has been achieved is necessary. Tabular representation of information is generally the best way to report data as it can be easily extracted by the user from an Excel or PDF file. Defining all quantities in such tables is very helpful to the user, i.e., the table should contain *all essential information*. The terminology, symbols, and proper reporting of results are not trivial and, it is critical that all reported and calculated quantities and metadata are clearly communicated so that they may be used by those reading the publication in future decades. Enzyme-catalyzed reactions are among the most important reactions occurring in living systems and, in particular, are critically important to metabolism and to the ties between genetic information and the expression thereof. Very substantial industrial use and economic impact also serve to increase the importance of these reactions. Thus, it is proposed that extensive thermodynamic network calculations be performed using existing thermodynamic property values for biochemical reactions with the aim of obtaining tables of modern, up-to-date standard molar formation properties for several thousands of biochemical substances and species, that are common to essentially all biological species. These calculations would also result in a reaction catalog [31] which would reveal discrepancies in the literature and could thus guide future experimental work.

## Appendix

The following Table lists the most important symbols with explanation and their units of measure.

**Table 1:** Symbols with their names and units.

Symbol	Name	Units	Notes

*a*	activity	1	
A_m_	Debye–Hückel constant, molality basis	kg^1/2^∙mol^−1/2^	
*c*	concentration	mol∙dm^−3^ (M)	
B	empirical constant in the extended Debye–Hückel equation	kg^1/2^∙mol^−1/2^	
*A*_r_	relative atomic mass	1	
*G*	Gibbs energy	J	
*G*′	transformed Gibbs energy	J	
*H*	enthalpy	J	
*I*	ionic strength	mol∙kg^−1^ or mol∙dm^−3^	
*k*_cat_	catalytic constant or turnover number	s^−1^	
*K*	equilibrium constant	1	^a^
*K*′	apparent equilibrium constant	1	^a^
*K*_M_ or *K*_P_	Michaelis constant	mol∙dm^-3^	
*M*_r_	relative molecular mass	1	
*m*	molality	mol∙kg^−1^	
*N*	number of a specified number of species in a system	1	
*p*	pressure	Pa	^b^
pCa	−log_10_[*a*(Ca^2+^)]	1	^c^
pH	−log_10_[*a*(H^+^)]	1	^d^
pMg	−log_10_[*a*(Mg^2+^)]	1	^c^
*Q*	reaction quotient	1	^a^
*Q*′	apparent reaction quotient	1	^a^
*R*	gas constant	J∙K^−1^∙mol^−1^	
*S*	entropy	J∙K^−1^	
*t*_1/2_	half-time	s	
*T*	thermodynamic temperature	K	
*x*	mole fraction	1	
*z*	charge number of an ion	1	
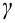	activity coefficient	1	
*ξ*	extent of a chemical reaction	mol	
*ξ*′	extent of a biochemical reaction	mol	
Δ_r_*G*	molar Gibbs energy of reaction	J∙mol^−1^	^e^
Δ_r_*G*′	transformed molar Gibbs energy of reaction	J∙mol^−1^	^e^
Δ_r_*G*°	standard molar Gibbs energy of reaction	J∙mol^−1^	^e^
Δ_r_*G*′°	standard transformed molar Gibbs energy of reaction	J∙mol^−1^	^e^
Δ*H*	enthalpy change	J	
Δ_r_*H*°	standard molar enthalpy of reaction	J∙mol^−1^	^e^
Δ_r_*H*′°	standard transformed molar enthalpy of reaction	J∙mol^−1^	^e^
Δ_r_*H*(cal)	calorimetrically determined molar enthalpy of reaction that includes the enthalpies of reaction of H^+^ and Mg^2+^ (consumed or produced) with any buffer in solution	J∙mol^−1^	^e^
Δ_r_*N*(H^+^)	change in binding of H^+^(aq) in a biochemical reaction	1	
Δ*Q*	change in reaction quotient	1	
Δ_r_*S*′°	standard transformed molar entropy of reaction	J∙K^−1^∙mol^−1^	^e^

^a^The value of an equilibrium constant will depend on the units and the standard state [19] when a reaction is not symmetrical. The symbols *K*_c_, *K*_m_¸ and *K*_x_ can be used, respectively, to denote the values of equilibrium constants based on concentration *c*, molality *m*, or mole fraction *x*. *K*_m_ should not be confused with the Michaels constant *K_M_*. Equilibrium constants can always be made dimensionless by insertion of a term such as 1 mol∙kg^−1^ or 1 mol∙dm^−3^. A dimensionless value of *K* is formally necessary if one wishes to calculate the logarithm of *K*. For this reason, we recommend the use of dimensionless *K*, *Q, K*′, and *Q*′. Similarly, the value of the reaction quotient *Q* of an unsymmetrical reaction will depend on the units used. Above all, *K, K*′, *Q*, and *Q*′ should be defined explicitly in order to specify the units in which concentrations are expressed and the standard states must also be specified. Conventions regarding *K, K*′, *Q*, and *Q*′ are dealt with in the body of this publication. ^b^1 Pa = 1 N m^−2^ = 10^−5^ bar. ^c^Values for pMg and pCa can be obtained by using a buffer for these metal ions or by adding a substantial excess of these metal ions over the species that bind them. Otherwise, values of pCa and pMg can be calculated by performing equilibrium modeling calculations [10]. ^d^The IUPAC definition of pH is *notional* in that, at present, there is no way to measure single-ion activities and the existing pH scale has been established by use of the Bates–Guggenheim convention [45]. Thus, when the pH has been obtained with a pH meter, one must use a value of 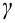
(H^+^) in order to calculate *m*(H^+^). An estimated value for 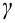
(H^+^) can be obtained by using a suitable Debye–Hückel type equation, even though this equation is imperfect for physiological ionic strengths. ^e^A subscript “m” is often used to denote molar quantities, e.g., use of 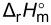 rather than Δ_r_*H*°.
